# Customizing galvanic vestibular stimulation amplitude: an objective technique using sway indices to account for individual sensitivity

**DOI:** 10.1007/s00221-026-07250-9

**Published:** 2026-03-10

**Authors:** Hailey A. Trier, Samantha B. Douglas, Jorge M. Serrador, Scott J. Wood

**Affiliations:** 1 Department of Psychology, Azusa Pacific University, Azusa, CA USA; 2 MARCS Institute for Brain, Behaviour and Development, Western Sydney University, Sydney, NSW Australia; 3 Neuroscience Laboratory, NASA Johnson Space Center, Houston, TX USA

**Keywords:** Balance, Threshold, Repeatability, Neuromodulation, Disorientation

## Abstract

Galvanic vestibular stimulation (GVS) uses transcutaneous direct current stimulation to either improve balance function at low intensities or provide a sensory disruption at higher intensities. The present study sought to establish an objective procedure for determining individual sensitivity levels for GVS using postural sway. GVS thresholds were obtained in healthy young adults over three sessions while standing with feet together and eyes closed using sinusoidal stimuli at intensity amplitudes ranging between 0.1 and 0.7 mA. Sway amplitudes from inertial motion sensors mounted on the head and torso were derived from sinusoidal fits over each 20 s trial. Individual sensitivities were determined using amplitude indices (lowest level that induced sway greater than sway without stimulation), critical indices using logistic analysis of sway amplitudes across the entire stimulus range, and subjective indices (lowest level that subjects perceived a sense of motion). Sensitivity indices for head and torso were strongly correlated, as were the corresponding amplitude and critical indices, and indices across multiple sessions. The sensitivity indices based on sway reflected greater intersubject variability than subjective indices. We conclude that a sinusoidally evoked postural sway sensitivity index provides an objective measure that may be used to account for intersubject GVS sensitivity.

## Introduction

While the potential effects of Galvanic vestibular stimulation (GVS) on balance have been known since the early 1800s (Dlugaiczyk et al. 2019), recent technological advances in neuromodulation have heightened interest in developing this technique for both clinical (Marchand et al. 2025) and aeromedical (Allred et al. 2024) applications. Transcutaneous delivery of direct current to the mastoid processes alters the continuous firing level of the peripheral vestibular afferents with minimal effects on other sensory systems (Fitzpatrick and Day 2004). Recent electrical stimulation studies have provided insight into the effects on both peripheral canal and otolith neural pathways, and central vestibular regions, offering insight for developing therapeutic approaches (Lopez and Cullen 2024).

The effects of GVS stimulus amplitude on a variety of outcome measures are well known and generally categorized as either subthreshold or suprathreshold (Dlugaiczyk et al. 2019; Lopez and Cullen 2024). At suprathreshold levels, GVS has been used to induce postural (MacDougall et al. 2006) and locomotion instability (Moore et al. 2006), as well as eye movement deviations (MacDougall et al. 2005). Suprathreshold levels have also been proposed for aeromedical applications to provide spatial disorientation training for pilots (Moore et al. 2011; Allred et al. 2024) or as a spaceflight analog of vestibular disruptions following g-transitions (Moudy et al. 2024). In contrast, subthreshold amplitudes, commonly referred to as noisy GVS, have been used to improve function in healthy, elderly, and patient populations (Wuehr et al. 2017; Stefani et al. 2020; McLaren et al. 2023). For example, at low intensities, GVS has been used to reduce postural sway (Mulavara et al. 2011; Goel et al. 2015; Fujimoto et al. 2016), increase the base of support during locomotion (Mulavara et al. 2015; Wuehr et al. 2016a), lower vestibular thresholds (Wuehr et al. 2023),increase ocular gain (Serrador et al. 2018) and produce cross-modal effects including the lowering of visual thresholds (Voros et al. 2021).

Since different levels of GVS result in opposite effects, it is important to characterize the optimal stimulus amplitude depending on one’s objective. One challenge is that an individual’s response to GVS can be affected by variables such as age (Jahn et al. 2003), repeated exposures (Balter et al. 2004; Moore et al. 2015), differences in electrode-tissue impedance (Okamoto et al. 2014) as well as other idiosyncratic factors (MacDougall et al. 2002). Thus, implementation of a predetermined stimulus amplitude to all subjects may not be equally effective for all individuals. Several implementations of GVS have adjusted stimulus amplitude according to the lowest levels that induce behavioral outcomes, but these methods have varied (see recent review in McLaren et al. 2023). Some studies have used subjective (e.g. verbal) reports of tingling (Wilkinson et al. 2008; Utz et al. 2011) or perception of body movement (Lobel et al. 1998). In other studies, subjective reports of motion have been captured with a joystick (Goel et al. 2015; Mulavara et al. 2015). Other studies have relied on observable sway either visually or with a recording, often in combination with subjective reports (Hlavacka et al. 1999; Pavlik et al. 1999; Bent et al. 2004; Goel et al. 2015; Mulavara et al. 2015; Samoudi et al. 2015; Wuehr et al. 2023). One limitation of the present literature is the lack of test-retest reliability measures.

Based on a recent review of GVS parameters needed for clinical neuromodulation applications (Valter et al. 2025), the use of individualized current intensity was recommended. The primary objective of the present study was to characterize objective sway-based indices that can be used to account for individual sensitivity. Three types of individual sensitivities were compared: (1) amplitude indices (lowest level that induced sway greater than sway without stimulation), (2) critical indices using logistic analysis of sway amplitudes across a stimulus range (0.1 to 0.7 mA), and (3) subjective indices (lowest level that subjects perceived a sense of motion). Inertial sensors on the head and torso were compared to determine the optimal sensor placement. Test-retest repeatability was examined by comparing results across three sessions. One goal of this research was to develop a robust technique that could be implementable with self-administered interventions using wearable technology.

## Materials and methods

### The participants

Twenty-nine healthy undergraduate students (age range 18–27 yrs, *M* = 20.8, *SD* = 2.3; 12 male, 17 female) completed this study for course credit. These 29 subjects included in the analysis ranged in height between 155 and 196 cm (*M* = 171.5, *SD* = 9.8) with body mass index range of 17 to 38 (*M* = 25.9, *SD* = 5.1). Exclusion criteria included recent injuries to the neck, back, or lower body; history of vestibular or neurological disorders; consumption of alcohol or other drugs affecting balance or alertness in the prior 24 h; and previous participation in studies involving GVS. One additional subject was excluded due to non-compliance with the narrow stance width required. In addition, participants were screened to ensure a general state of good health prior to each session. Participants gave written informed consent before participating, and all test procedures were approved by the university’s Institutional Review Board.

### Equipment

Participants wore inertial motion sensors (MTi, Xsens Technologies, The Netherlands) on the torso and head. The head sensor was centered over the crown using a pivoting platform to level the sensor during upright stance prior to GVS stimulus onset. The torso sensor was mounted near the center of gravity on an adjustable velcro strap fit snugly around the waist that also held a wireless Xsens transmitter. The sensors were oriented so that medial-lateral (ML) sway was obtained from roll orientation and anterior-posterior (AP) sway was obtained from pitch orientation, both with an angular resolution of 0.05 deg. Three-dimensional orientation data was wirelessly transmitted to a custom Linux-based data acquisition system using LabWindows CVI (National Instruments, Austin TX) and sampled at 100 Hz along with the GVS stimulus data. Sinusoidal GVS was supplied by a portable current stimulator with subject isolation that has been previously described (Mulavara et al. 2011).

Prior to placement of electrodes, skin at the electrode site was cleaned with skin prep gel and alcohol prep wipes. A conductive gel was applied to the skin/electrode interface to reduce contact impedance and to alleviate discomfort. Stimulation was administered through two ear-clip electrodes affixed to the earlobes (Alpha-Stim, Electromedical Products International, Inc., Mineral Wells, TX). A measure of electrode contact impedance was obtained to ensure consistency in electrode preparation prior to testing (*M* = 1.91 kΩ, *SD* = 1.16 kΩ). A bipolar sinusoidal stimulus with frequency of 0.1 Hz was applied bilaterally across electrodes. Coats (1972) had previously demonstrated that sway during sinusoidal GVS was most observable at frequencies ranging from 0.025 Hz to 0.200 Hz and peaked at 0.1 Hz. This frequency is within a range that the otoliths play a major role in modulating postural responses and body segments move en bloc above the ankle joint (Nashner et al. 1989). The GVS profiles were saved to the portable stimulator memory card and output at 100 Hz, initiated by the operator after the subject assumed a stable upright posture with eyes closed according to the protocol described below.

### Procedure

Testing was conducted during three one-hour sessions in the same week typically with one day in between each session. During the first session, a medical history questionnaire was used to review exclusion criteria with each subject, and a pretest questionnaire was used each session to verify daily health status prior to testing. After applying the electrodes, the motion sensors were donned and manually leveled relative to gravity, and their orientation was visually verified by the operator by monitoring the signal output during voluntary movements in different planes. Subjects were instructed to stand upright on a solid surface with their feet together to minimize the ML base of support. Participants wore socks only and maintained elbows at their sides while holding a lightweight foam board with both hands. Prior to closing their eyes at the start of each trial, subjects viewed a visual target at 3 m distance aligned with straight ahead gaze to ensure that head orientation remained consistent across trials, thus ensuring that the sway responses would remained predominantly aligned with both head interaural and torso medial-lateral axes (Fitzpatrick and Day 2004). During the trials participants were instructed not to raise or lower the chin nor bend at the waist or knees. Subjects were informed about the purpose of the electrical stimulus to elicit sway and were instructed to not resist any compulsion to sway. Subjects were also instructed to minimize head movements during the trials so that their head and torso swayed en bloc. A research assistant stood behind the participant ready to provide assistance in case of a loss of balance, which was intended to give participants a greater sense of safety when experiencing postural sway at the higher stimulus levels. Criteria used to terminate trials due to loss of balance included when subjects moved their feet, began to take a step, or raised their arms.

Except for the first three subjects tested, participants were asked to provide a verbal report if they detected the onset of a subjective sense of motion during the trials. Instructions before each trial specifically requested that the verbal cue be given at the onset of a sense of motion attributable to the electrical stimulus independent of self-induced sway or tactile sensations at the earlobes. The lowest stimulus amplitude at which this self-report of motion was given was considered the subjective index for that session. Generally, all higher stimulus amplitudes greater than this subjective index also evoked perceptual reports of motion.

Each GVS profile lasted 125 s and consisted of five stimulus amplitudes (20 s each, 2 cycles of 0.1 Hz) presented in either increasing or decreasing fashion interspersed with 5-second periods of zero stimulation. The zero-stimulation intervals were intended to record baseline postural sway, and to minimize carryover effects from one level of stimulation to the next. Subjects had no prior experience with GVS stimulation. While they were not provided any familiarization trials, the stimulus progression always started at the lowest level. Trials overlapped by 0.2 mA; for example, the first trial progressed from 0.1 to 0.5 mA and the second trial progressed from 0.3 to 0.7 mA. Although over 70% of the sessions included levels higher than 0.7 mA (0.5–0.9 mA and 0.7–1.1 mA ranges), for consistency in subsequent analyses stimuli greater than 0.7 mA were excluded since none of the sensitivity indices exceeded 0.7 mA. Four subjects received additional stimulus profiles that included smaller increments than 0.1 mA, and these were also excluded from subsequent analysis for consistency across all subjects. In between the GVS profiles participants were allowed to sit down to rest. During these breaks, they were asked to rate discomfort at the site of the electrodes from 0 being ‘no irritation’ to 4 being ‘very irritating/almost painful’; and to describe the sensation of motion and/or disorientation produced by the stimulation as well as any adverse symptoms. The testing was terminated with self-reports of mild motion sickness symptoms or discomfort at the electrode site, which were more prevalent at stimulus levels greater than 0.7 mA.

### Data analysis

**Fig. 1:**
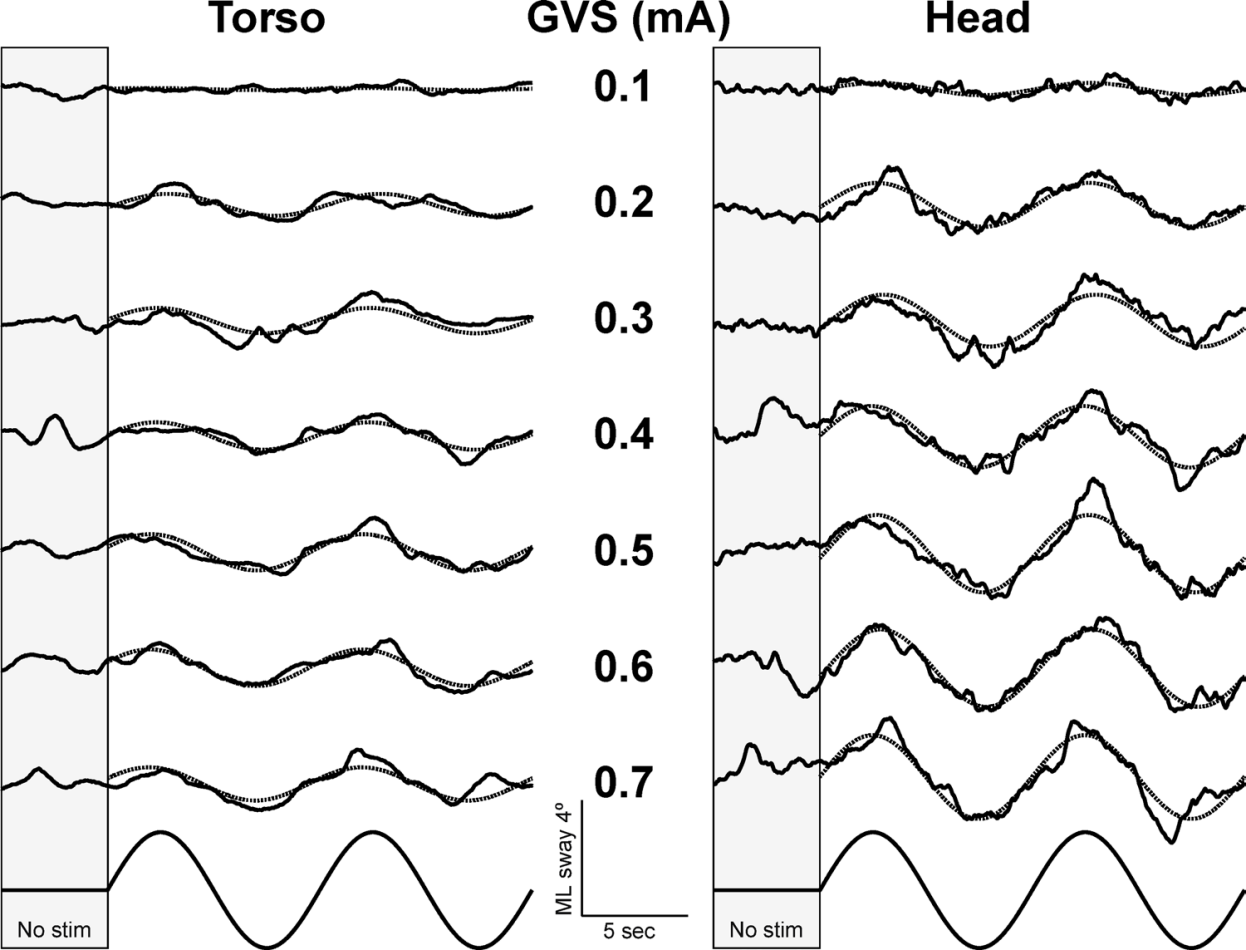
Comparison of ML sway in one subject’s session for inertial sensors located on the torso (left) and on the head (right) across trials with increasing GVS amplitude. At the beginning of each trial, sway was recorded for 5 s with no stimulation. The amplitude of sway during the stimulation period for each trial was derived from sinusoidal curve fits (dotted lines). The timing of the stimulus is shown on the bottom trace, and the scale is the same for torso and head

Postural sway was analyzed separately for ML and AP directions, and separately for the torso and head sensors. Figure 1 illustrates an example of ML sway in one session for both sensors across the stimulus range of 0.1 to 0.7 mA. The average number of trials for each session was 27 ± 7 (M ± SD) across all subjects ranging between 1 and 6 repetitions at each stimulus level. To control for uneven number of trials at each stimulus level, a median response amplitude from the sinusoidal curve fits was calculated for each stimulus level from each session. The use of a median measure had the added benefit of minimizing the impact of outlier data points. Due to the narrow stance width, ML sway response was modulated as a function of stimulus amplitude (Fig. 1) while AP sway did not consistently vary systematically with stimulus amplitude. Therefore, only ML sway was used for calculation of the sensitivity indices.

Nonlinear least squares sinusoidal curve fits (Matlab, MathWorks Inc) were used to characterize the modulation of sway as a function of the sinusoidal-varying stimuli. Sway amplitude and phase were derived from the curve fits at the known stimulus frequency of 0.1 Hz. Since confidence limits for phase measures are inversely related to the amplitude, becoming inherently noisy around threshold response levels, we chose to focus our analyses on sway amplitude as our primary outcome measure. Root-mean-square (RMS) sway was also calculated for each 5 s block prior to stimulation, and the median of these RMS scores was used as the sessions baseline sway separately for both sensors.

**Fig. 2:**
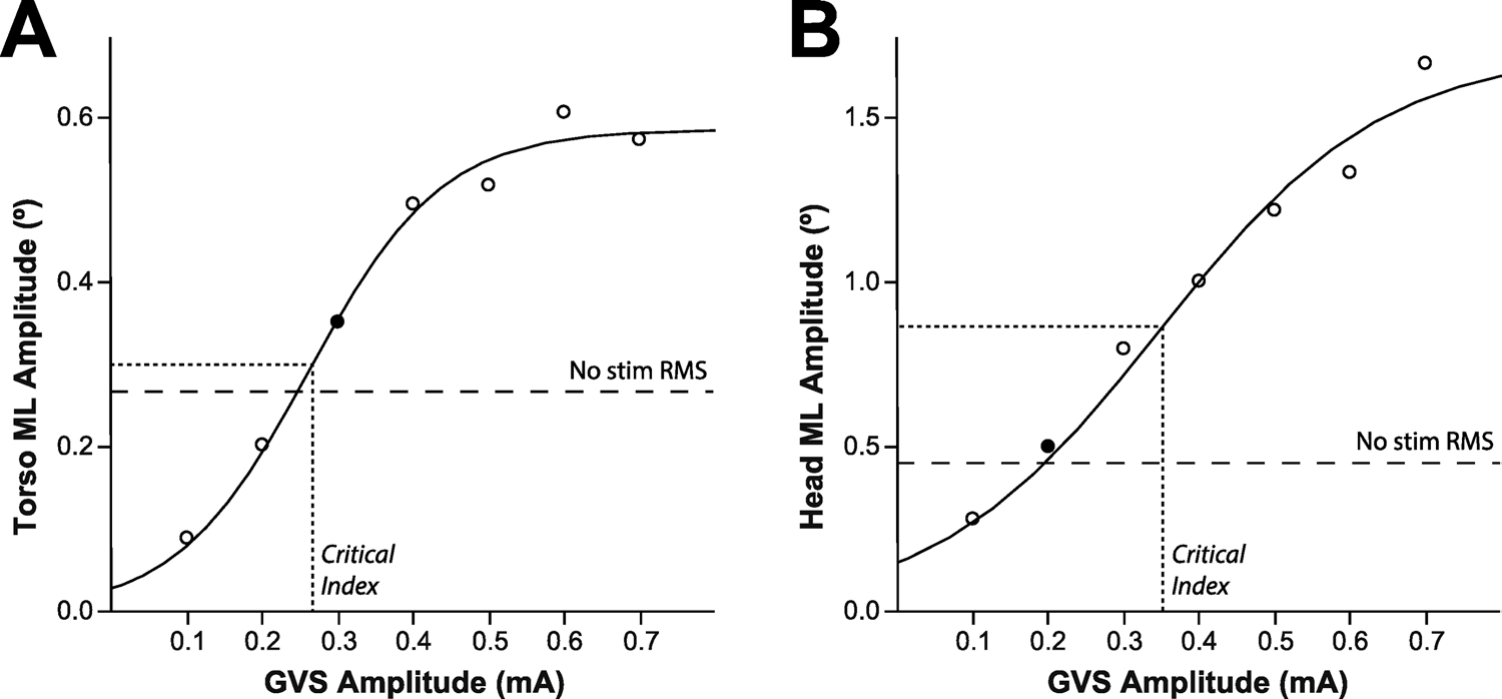
Example of the ML sway based sensitivity indices calculated from both torso (**A**) and head (**B**) sensors (same session data as shown in Fig. 1). The amplitude indices (filled circles) were based on the lowest stimulus amplitude that evoked sinusoidal sway amplitude greater than the median RMS sway (dashed lines) captured from the no stimulus periods at the beginning of each trial. The critical indices (dotted lines) were derived from the logistic 3P fits of the sinusoidal sway amplitude evoked over the entire range of stimulus levels used for these fits (0.1–0.7 mA)

These median sway measures were then used to calculate two types of sensitivity indices for each session (Fig. 2). The first was a threshold-type amplitude index (a.i.) defined as the lowest stimulus level where the sinusoidal response amplitude exceeded the baseline RMS sway amplitude. The second critical index (*ci*) was derived from a logistic 3-parameter fit model using responses at all seven levels and assuming a negligible sinusoidal response pattern with no stimuli (JMP Pro, SAS Institute, Inc). The logistic 3P curve was chosen to characterize the point of inflection where the observed change in sway was greatest with increasing stimulus amplitudes. At low stimulus amplitudes, the sway was negligible and did not exceed natural sway occurring without a stimulus. As the stimulus amplitude increased, there was a corresponding increase in sway that often reached an asymptote. Although subjects were instructed not to resist sway, this asymptote is a function of participants’ compensatory strategies when approaching one’s limits of stability during GVS-induced sway. Above 0.7 mA sway would occasionally decrease due to these compensatory strategies. For subjects with greater GVS sensitivity (thresholds typically < 0.15 mA), the higher stimulus values maybe more prone to amplitude fluctuations from these types of compensatory strategies. For some of these sessions, it was necessary to delete the higher stimulus value data points to converge on the expected fit within the 0.1–0.7 mA range. As opposed to the amplitude indices, this critical index has the advantage of being derived from a curve fit over the entire range of responses. This curve-fit based index also allows for an interpolation between stimulus levels used.

Non-normal distributions for both sway amplitude and all sensitivity indices were confirmed using Shapiro-Wilk test; therefore, non-parametric tests were required for all statistical analyses. Comparisons of multiple sessions and stimulus levels used Friedman’s Analysis of Variance for repeated measures while paired comparisons used Wilcoxon Signed Ranks test. Kendall’s tau (τ) correlation coefficients were calculated to examine relationships between the various sensitivity indices. This statistic was chosen over the similar Spearman’s rho due to the small sample size and the fact that many of the amplitude indices across sessions were the same value.

## Results

### Sway responses

While there were clear differences in GVS sensitivity across subjects, all 29 participants were able to complete trials without loss of balance during sinusoidal GVS stimulation up to ± 0.7 mA with eyes closed on stable support surface and narrow stance. The range of responses from the torso sensor suggested that all subjects’ center of gravity remained within a functional stability region over this stimulus range, while head sway amplitude occasionally exceeded a theoretical maximum of 8 deg laterally without resulting in a loss of balance (Holbein and Chaffin 1997). As described in Sect. "Procedure", the GVS profiles started at the lowest level and progressed to higher ranges to account for individual variability in what amplitudes may be tolerated. Stimulus ranges exceeding 0.7 mA resulted in either adverse side effects (see Sect. "Adverse symptoms" below) and/or loss of balance in 30% of the subjects. In these cases, trials with higher stimulus ranges (0.5–0.9 mA and 0.7–1.1 mA) were either discarded or not completed. For completed trials above 0.7 mA in the remainder of the subjects, the postural responses were often more variable presumably due to compensatory responses in order to prevent loss of balance. We were unable to derive the critical index in approximately 5% of the sessions due to this increased variability. For consistency across subjects and to minimize the effects of increasing compensatory responses at higher stimulus levels, we elected to discard data above 0.7 mA.

**Fig. 3:**
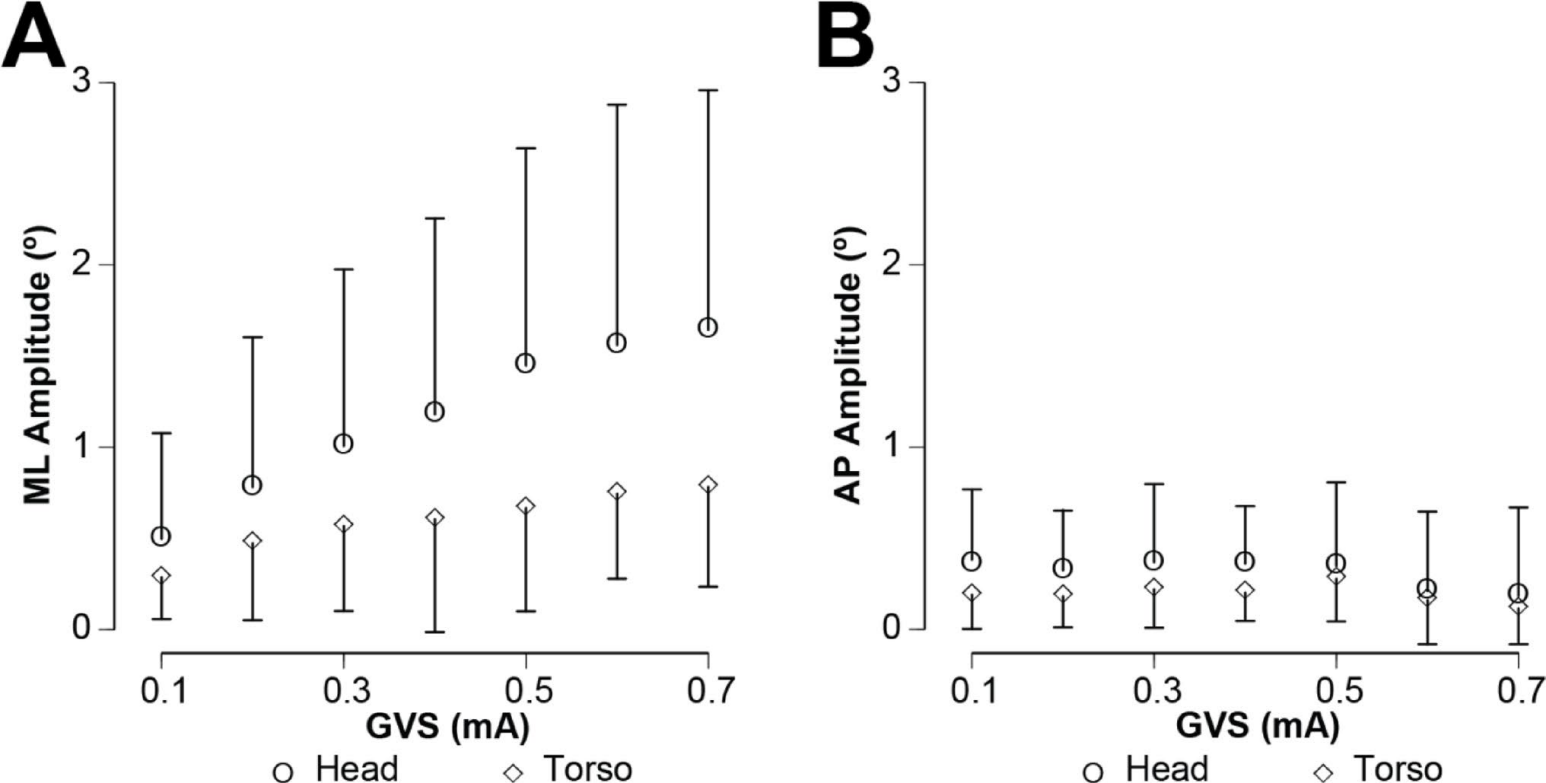
The median amplitude of ML sway (**A**) and AP sway (**B**) across all subjects (*n* = 29) is plotted for both torso (diamond) and head (circle) sensors across all levels of GVS. Error bars reflect the interquartile ranges. There was a significant effect of stimulus level for both head and torso ML sway, and the amplitudes of the head movements were significantly greater than torso movements across all stimulus amplitudes. In contrast, the variation in AP sway as a function of stimulus level was negligible

As illustrated in Fig. 1, the ML sway responses at both head and torso were primarily in phase with the stimulus at this 0.1 Hz frequency, although the movements were consistently greater at the head. Based on Friedman’s ANOVA, there was a significant effect of stimulus level between 0.1 and 0.7 mA on both torso (χ^2^ (6, *n* = 87) = 223.8, *p* < 001) and head ML sway amplitude (χ^2^ (6, *n* = 87) = 278.4, *p* < 0.001, Fig. 3). ML sway amplitude at the head was significantly greater than the torso (Wilcoxon Signed Rank, Z = − 21.1, *p* < 0.001). There were, however, no significant differences in sway amplitude across the three sessions.

### Comparison of sensitivity indices

**Fig. 4:**
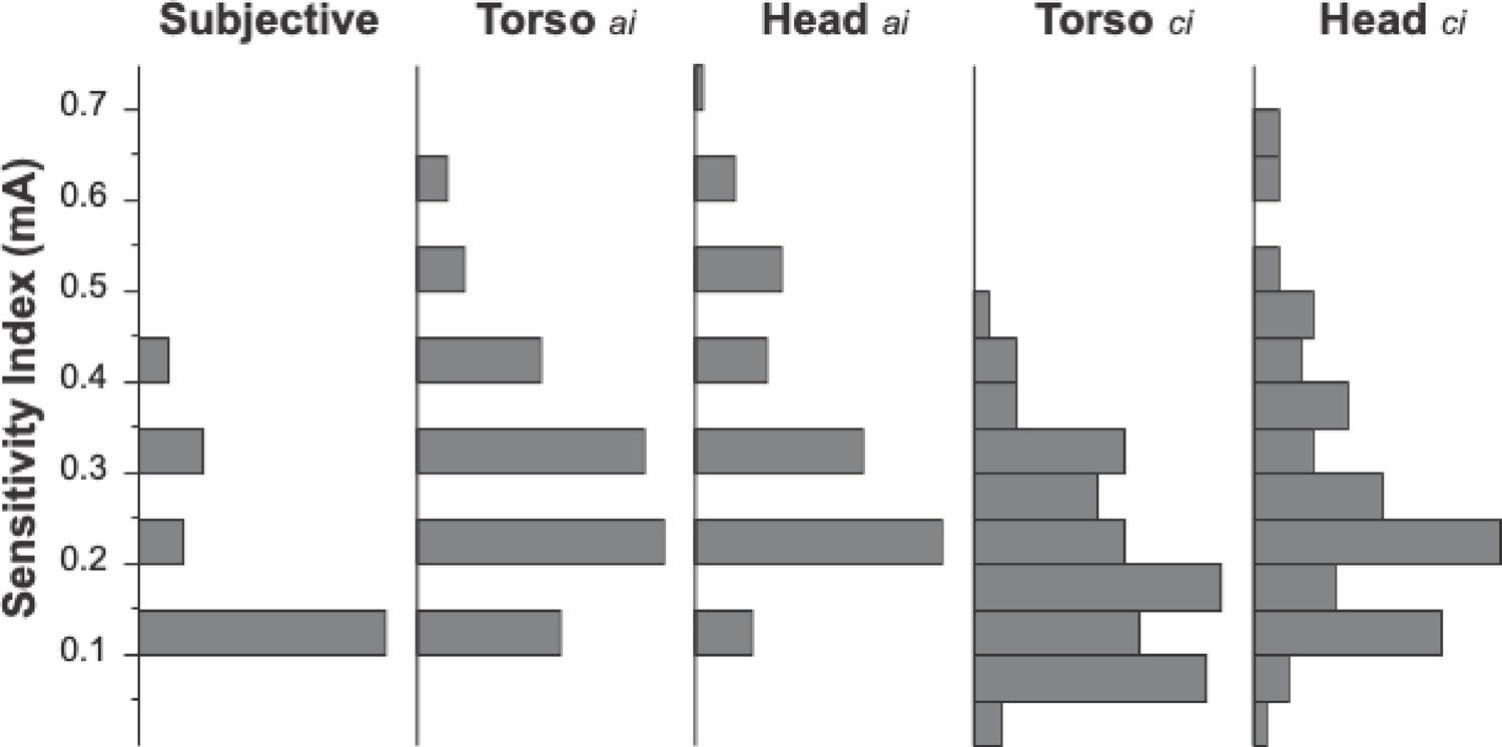
The distributions are presented for subjective indices from verbal reports, the amplitude indices (a.i.) from torso and head sway, and the logistic fit based torso and head critical indices (ci). Note the resolution of subjective and amplitude indices are limited to the stimulus levels tested, while the logistic based fits allow for an interpolation between levels tested

The distributions of all sway indices were rightward skewed with the subjective indices significantly smaller than the sway-based indices (Wilcoxon Signed Rank Test, Torso a.i.: Z = − 5.0, *p* < 0.001, Head a.i. Z = − 5.8, *p* < 0.001, Torso ci Z = − 2.6, *p* = 0.008, Head ci Z = − 4.9, *p* < 0.001). As illustrated in Fig. 4, the majority of the subjective indices (64%) were at the smallest level tested. The median for the subjective amplitude index was 0.1 mA for each of the three sessions, with an overall interquartile range (IQR) of 0.2 mA. Twelve of 29 participants reported a sensation of sway immediately at the lowest amplitude of stimulation for every session. As stated in Sect. "Procedure", all higher stimulus amplitudes greater than this subjective index also evoked perceptual reports of motion. Six participants reported a feeling of sway before the onset of stimulation in at least one trial.

The amplitude indices for the torso and head sensor were intended to provide an objective, sway-based equivalent to the verbal reports. Across all three sessions, the median for both of these indices was 0.2 mA with a 0.3 mA IQR. The values for the torso critical index were the closest to the verbal amplitude index (0.19 mA median and 0.15 mA IQR). In contrast, the distribution for the head critical indices had a median value of 0.25 mA with an IQR of 0.2 mA. The critical index was below 0.1 mA in 22% of the sessions for the torso sensor versus less than 5% of the sessions for the head sensor.

### Test-retest reliability

Due to the skewed distributions, test-retest reliability was evaluated in part using non-parametric correlations between sessions 1 and 2, between sessions 2 and 3 and between sessions 1 and 3. The subjective indices were positively correlated across all sessions, with Kendall’s τ ranging between 0.48 and 0.90 (all *p* < 0.01, two-tailed). This would be expected given the high percentage of measures at 0.1 mA. The amplitude indices for both torso and head sensors were also significantly correlated, with Kendall’s τ ranging between 0.30 and 0.48 (*p* values range from 0.05 to < 0.01). The correlation coefficients for the critical indices exhibited less agreement, with Kendall’s τ ranging between 0.22 and 0.44 for the torso sensor (*p* values range from 0.10 to < 0.01) and between 0.18 and 0.33 for the head (p value range from 0.18 to 0.01).

**Fig. 5:**
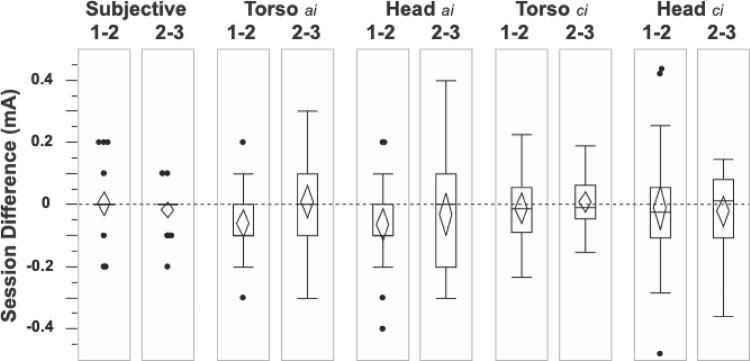
To assess repeatability across sessions, differences were calculated for each individual between sessions 1 and 2 and then between sessions 2 and 3 for each sensitivity index. The distributions of these session differences are represented by the box plots where the box represents the interquartile range, the horizontal line is the median score, and the whiskers are the 5th and 95th percentiles. The mean and its corresponding confidence interval are represented by the diamond. The high repeatability of the verbal measures stems from the fact that 64% of these indices were at the lowest stimulus tested (0.1 mA)

Test-retest repeatability was also examined by calculating difference scores across sessions (Fig. 5). Similar to sway amplitude scores (see "Sway responses" above), Friedman’s ANOVA resulted in no significant differences detected for the verbal or logistic-based critical indices. There was a significant decrease in the sway-based amplitude indices between sessions 1 and 2 for both the torso sensor (Z = − 2.5, *p* = 0.01) and head sensor (Z = − 2.2, *p* = 0.03, Wilcoxon Signed Rank Tests). While the inclusion of a familiarity trial would minimize this, it is important to note that the indices were repeatable across all sessions with the majority of session differences (> 70%) not exceeding 0.1 mA.

### Comparison of different types of sensitivity indices

**Fig. 6:**
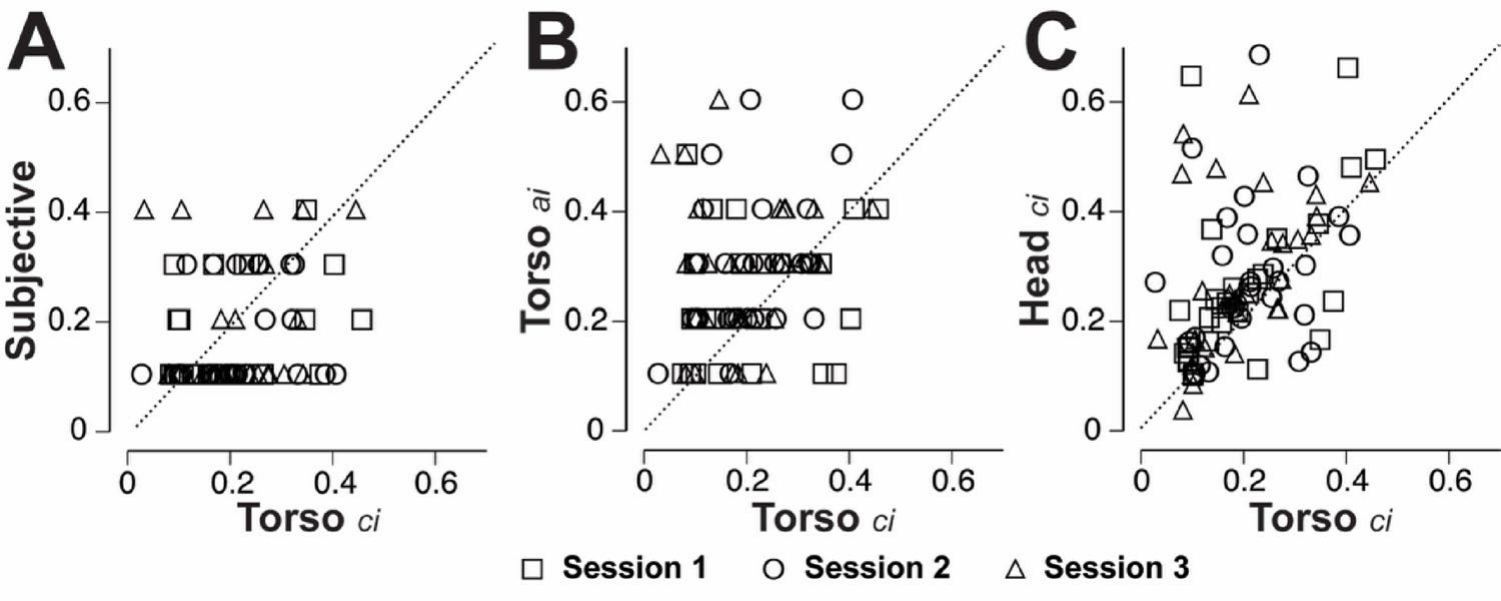
Scatterplots with the Torso critical indices (ci) are shown to illustrate the relationship between objective and subjective measures (**A**, verbal amplitude indices), the relationship between logistic and amplitude based sway indices (**B**, torso amplitude indices, or a.i.) and the relationship between torso and head amplitude indices (**C**, head ci). The dotted unity lines represent a perfect correspondence for each comparison. All values are in mA

Since the goal of these methods is to customize the level of GVS to account for individual sensitivity, it is interesting to compare the relationships between the different types of sensitivity indices. Figure 6 illustrates three types of comparisons while separating out the three sessions using different symbols. As described above, the prevalence of the lowest level (0.1 mA) for the verbal amplitude index in our study may limit its value in differentiating individual sensitivity. The verbal amplitude indices generally showed a weak positive relationship with the sway-based measures, only reaching significance with the torso critical indices (τ = 0.23, *p* = 0.009). When examining this relationship in Fig. 6A, one can observe a relatively wide range of torso critical indices (0.02–0.4 mA) that corresponded with a verbal amplitude index of 0.1 mA.

The method of deriving the amplitude indices was chosen to rely on an objective sway based measure using a similar strategy to what subjects were instructed to do with the subjective reports, namely identify the lowest level where GVS induced sway exceeded natural sway patterns. This resulted in a wider range of responses than the verbal reports, and sway amplitude indices were generally well correlated with the corresponding sway-based critical indices. For example, Fig. 6B illustrates the association between the Torso amplitude and critical indices (τ = 0.22, *p* = 0.007). The Head amplitude and critical indices were also significantly correlated (τ = 0.35, *p* < 0.001).

It is also interesting to note the relationship between the indices obtained with the inertial sensor mounted on the head versus the torso. While these data could have been combined into a multi-segment model, we chose to keep these separate for the purpose of addressing the potential for using a single sensor location for future self-administered type protocols. While the ML sway amplitude was larger at the Head than the Torso, the corresponding indices remained well correlated with each other. Figure 6C illustrates the relationship between the Head and Torso critical indices (τ = 0.37, *p* < 0.001). The Head and Torso amplitude indices were also significantly correlated (τ = 0.38, *p* < 0.001). While significant, the weakness of these correlations illustrated in Fig. 6 reflect the inherent variability of the responses and complexities in these relationships.

### Adverse symptoms

Subjective data collected in-between trials included participants’ rating of electrode discomfort and descriptions of any other adverse symptoms, prompted in an open-ended manner so as not to influence responses. More common adverse symptoms included tingling, itching, pain (e.g., stinging) at the electrode site, and less common symptoms included dizziness or nausea. The presence of each was marked in a binary manner according to whether the adverse symptom was reported. Not surprisingly, adverse symptoms were more prevalent at stimulus amplitudes greater than 0.7 mA, which could be attributable to relatively small surface area of the ear clip electrodes used. When considering only the 0.1–0.7 mA range used to determine the sensitivity indices, there was no effect of session number on electrode discomfort severity. In addition, the incidence of adverse symptom reporting did not differ by session (all *p* > 0.05, two-tailed Fisher’s exact test). Of all 29 participants tested, only 1 requested to stop the experiment, citing discomfort at the electrode site.

## Discussion

The goal of this study was to characterize an objective procedure for determining individual sensitivity levels for GVS using sinusoidally-induced postural sway. We demonstrated that in healthy subjects while standing on a fixed support with eyes closed and narrow stance, sinusoidal GVS at 0.1 Hz induces increasing ML sway between 0.1 and 0.7 mA stimulus levels that can be used to objectively derive sensitivity indices. Our results suggest that a single inertial sensor located near the subject’s center of gravity on the torso or alternatively located on the head was sufficient to derive these sway-based indices. We propose that this technique would be implementable in neuromodulation applications that would benefit by optimizing GVS stimulation levels to account for differences in sensitivity.

While thresholds based on cutaneous sensations at the electrode site have been effective in dynamic treadmill walking (e.g., Wuehr et al. 2016b), our subjects during the standing balance trials were specifically instructed to report when they perceived a sense of motion versus the presence of tactile cues at the electrode site. These subjective indices were routinely lower than sway-based measures, with the majority at the lowest stimulus amplitude tested. The presence of false positives (in which participants reported feeling a sense of sway greater than normal they attributed to the stimulus before the onset of stimulation) further characterized the idiosyncratic nature of these perceptual measures. This underscores the potential benefits of an objective behavioral measure to optimize individual sensitivity for neuromodulation applications.

We chose to utilize commercially available ear clip electrodes that had prior approval for clinical neuromodulation applications (Brunye et al. 2021). Since these electrodes have a successful history of patient self-administration in prior clinical trials using subthreshold stimuli, we anticipated that developing a customization procedure using this electrode configuration would facilitate transition to future clinical field trials using GVS. It should be noted that this stimulation through the ear lobes deviates from the traditional mastoid electrode placement used in prior bilateral-bipolar GVS investigations (Marchand et al. 2025). While both the reported perceptual (sense of roll rotation) and sway responses (in the direction of the anode) were consistent with prior GVS studies, this electrode placement certainly impacted the current flow to peripheral vestibular system (Truong et al. 2024) and may stimulate central pathways differently than transmastoid applied GVS (Marchand et al. 2025). Future GVS studies with this ear clip configuration could measure postural responses with different head orientations (e.g., during head turns and tilts) as further validation of this craniocentricity of the responses (e.g., Fitzpatrick and Day 2004; Khosravi-Hashemi et al. 2019).

It should be noted that the sensitivity indices we observed were lower than GVS thresholds reported elsewhere in the literature. This may in part result from the unique electrode features used (Truong et al. 2023) as well as our healthy young subject cohort. As reviewed by McLaren et al. (2023), similar motion-based thresholds in healthy subjects using transmastoid stimulation are typically in the 0.5–0.6 mA range, and generally higher in patients with bilateral vestibulopathy. The specific GVS waveforms are another factor; and our stimulus frequency of 0.1 Hz would be expected to result in a lower sensitivity index. For example, using a direction discrimination task Peters et al. (2015) reported GVS thresholds varied between 0.7 mA at 0.05 Hz to 1.4 mA at 2 Hz. Not surprisingly, thresholds also vary by the behavioral outcome used to detect changes with stimulus intensity. Using GVS step profiles between 0.5 and 2 mA, Nguyen et al. (2022) found subjective thresholds around 0.9 mA versus GVS-induced oculomotor thresholds around 1.6 mA. Aside from the imprecision in deriving these thresholds, it is reasonable to assume there is an amplitude range of near “subthreshold” GVS stimuli that may entrain behavior similar to suprathreshold stimuli. Noisy GVS studies using an amplitude of 50% of motion perception thresholds have generally been more effective at improving postural control than others using 80–100% threshold stimuli (McLaren et al. 2023). Our results are consistent with Assländer et al. (2021) who concluded that noisy GVS in young healthy subjects with intact vestibular function should be targeted at stimulus amplitudes below the 0.4–0.6 mA estimated peripheral thresholds (Kwan et al. 2019) to minimize postural compensatory responses.

The sway-based amplitude index is an efficient means of incorporating an objective measure. After establishing a participant’s baseline sway without stimulation, the amplitude indices offer the conceptual advantage of not requiring testing beyond the lowest level that induces sway greater than the baseline (e.g., Inglis et al. 1995). The curve-fit based critical indices, on the other hand, offer the benefits of determining sensitivity using a curve fit over a targeted stimulus range that allows interpolation between the stimulus levels used. One of the challenges of this approach relates to the saturation of sway at higher stimulus amplitudes and increased sway variability at these higher intensities. This phenomenon was previously observed in a study by Latt et al. (2003) in which magnitude of sway responses to sinusoidal GVS increased as the amplitude of the stimulus increased from 0.05 to 0.5 mA then saturated at higher stimulus amplitudes.

At higher stimulus amplitudes, we observed more variability in sway that complicated the determination of the critical indices from the logistic fits. We were unable to derive the critical index in approximately 5% of the sessions due to this increased variability. This increased sway in our healthy cohort at higher stimulus amplitudes may represent the introduction of non-linear compensatory control strategies that have been demonstrated with narrow stance and external perturbations (Goodworth et al. 2014). Similar compensatory strategies in balance impaired populations (Horak 2006) may also complicate the implementation of logistic-fit based thresholds in populations that are targets of GVS-based neuromodulation. As reviewed by McLaren et al. (McLaren et al. 2023), underlying vestibular pathology may also result in higher thresholds requiring greater stimulus amplitudes. The eyes closed and narrow stance methodology was intended to remove vision and decrease the lateral base of support while maximizing the sensitivity of vestibular-induced postural sway. Modifications to the task (e.g., wider stance or eyes open) may be necessary in applying a logistic-fit based approach with balance-impaired populations.

Previous GVS studies have used thresholding techniques based on similar verbal reports of perception (Lobel et al. 1998; Wilkinson et al. 2008; Utz et al. 2011), observation of body sway (Pavlik et al. 1999; Samoudi et al. 2015), or both (Inglis et al. 1995; Bent et al. 2000). Results from the present study indicate that although both types of measures can show good test-retest reliability, the sway based measures resulted in greater intersubject variability. Given the challenges of the logistic fits, the amplitude index based on increased body sway may be preferred to generalize to clinical implementation. Since the amplitude indices only require levels that induce sway greater than baseline, this method also offers more efficiency to achieve an outcome without added fall risk (McLaren et al. 2023).

The application of our approach may be limited to healthy young subjects as this sway-based approach may need to be adapted with impaired populations such as bilateral vestibulopathy who would have more pronounced baseline sway and fall risk with a narrow stance and eyes closed. The selection of a 0.1 Hz waveform was chosen based on prior studies demonstrating sinusoidal GVS induced sway peaked at this frequency (Coats 1972). However, sensitivity to a sinusoidal waveform may not translate to other stimulus waveforms such as the broad-band frequency typically utilized for noisy GVS applications. Another limitation is the unique ear-clip electrodes that were utilized that are typically limited to stimulus amplitudes of 0.6 mA or lower (Brunye et al. 2021). Future work should explore how these sensitivity indices are influenced by different electrode configurations, randomized stimulus presentation order, and using different types of GVS waveforms (e.g., steps or broad-band noise). Future directions for this research include the development of handheld technologies for at-home thresholding in combination with clinical GVS applications such as stochastic noise. Further investigation of the relationship between perceptual and sway thresholds could also be useful in fine-tuning the thresholding procedure.

## Conclusions

We conclude that in young healthy subjects a sinusoidally evoked postural sway sensitivity index provides an objective measure that may be used to account for intersubject GVS sensitivity. If these indices prove useful for optimizing the stimulus level for clinical interventions, such as subthreshold stimuli, then this approach may also be used to track changes in GVS sensitivity when these interventions are implemented over time. For other suprathreshold applications, these indices may also prove useful to characterize GVS sensitivity for between-subjects experimental designs. One important feature of our protocol was utilizing a narrow stance so that the primary sway was in the ML direction. Utilizing a 0.1 Hz stimulus was efficient to reliably capture lateral sway at low stimulus levels where sway was primarily about the ankles with head and torso in synch with the stimulus profile. Reliable indices can be obtained using sensors at the head or torso, and using simple amplitude based criteria or more complex logistic curve fits. While the logistic based torso critical indices were correlated with verbal indices, the wider range of responses of the sway-based measures reflect differences between subjective and objective approaches to capture the individual variability in GVS sensitivity.

## Data Availability

The data that support the findings of this study are available from the authors upon reasonable request.
